# Enterovirus 71–associated Hand, Foot, and Mouth Disease, Southern Vietnam, 2011

**DOI:** 10.3201/eid1812.120929

**Published:** 2012-12

**Authors:** Truong Huu Khanh, Saraswathy Sabanathan, Tran Tan Thanh, Le Phan Kim Thoa, Tang Chi Thuong, Vu thi Ty Hang, Jeremy Farrar, Tran Tinh Hien, Nguyen van Vinh Chau, H. Rogier van Doorn

**Affiliations:** Author affiliations: Children’s Hospital 1, Ho Chi Minh City, Vietnam (T.H. Khanh, L.P.K. Thoa, T.C. Thuong);; Oxford University Clinical Research Unit, Oxford, UK (S. Sabanathan, T.T. Thanh, V.t.T. Hang, J. Farrar, T.T. Hien, H.R. van Doorn);; Oxford University (S. Sabanathan, J. Farrar, T.T. Hien, H.R. van Doorn);; Hospital for Tropical Diseases, Ho Chi Minh City (N.v.V. Chau)

**Keywords:** hand foot and mouth disease, Vietnam, enterovirus 71, viruses, children, enterovirus

## Medscape CME Activity

Medscape, LLC is pleased to provide online continuing medical education (CME) for this journal article, allowing clinicians the opportunity to earn CME credit. 

This activity has been planned and implemented in accordance with the Essential Areas and policies of the Accreditation Council for Continuing Medical Education through the joint sponsorship of Medscape, LLC and Emerging Infectious Diseases. Medscape, LLC is accredited by the ACCME to provide continuing medical education for physicians.

Medscape, LLC designates this Journal-based CME activity for a maximum of 1 *AMA PRA Category 1 Credit(s)*^TM^. Physicians should claim only the credit commensurate with the extent of their participation in the activity.

All other clinicians completing this activity will be issued a certificate of participation. To participate in this journal CME activity: (1) review the learning objectives and author disclosures; (2) study the education content; (3) take the post-test with a 70% minimum passing score and complete the evaluation at www.medscape.org/journal/eid; (4) view/print certificate.

**Release date: November 16, 2012; Expiration date: November 16, 2013**

## Learning Objectives

Upon completion of this activity, participants will be able to:

- Distinguish the virus type associated with the outbreak of hand, foot, and mouth disease (HFMD) in Vietnam in 2011
- Analyze how to grade the clinical severity of HFMD
- Evaluate the clinical presentation of HFMD in the current report
- Assess the management of HFMD

## CME Editor

**Karen L. Foster, **Technical Writer/Editor, Emerging Infectious Diseases. *Disclosure: Karen L. Foster has disclosed no relevant financial relationships.*

## CME Author

**Charles P. Vega, MD, **Health Sciences Clinical Professor; Residency Director, Department of Family Medicine, University of California, Irvine. *Disclosure: Charles P. Vega, MD, has disclosed no relevant financial relationships.*

## Authors

*Disclosures:* ***Truong Huu Khanh, MD*** *;* ***Saraswathy Sabanathan, MD*** *;* ***Tran Tan Thanh, PhD*** *;* ***Le Phan Kim Thoa, MD*** *;* ***Tang Chi Thuong, MD, PhD*** *;* ***Vu thi Ty Hang, PhD*** *;* ***Jeremy Farrar, BSc, MBBS, FRCP, DPhil*** *;* ***Tran Tinh Hien, MD, PhD*** *;* ***Nguyen van Vinh Chau, MD, PhD*** *; and* ***H. Rogier van Doorn, MD, PhD,*** *have disclosed no relevant financial relationships.*
