# Supplementary material for: Enterovirus 71–associated Hand, Foot, and Mouth Disease, Southern Vietnam, 2011
Source: Emerg Infect Dis. 2012 Dec;18(12):2002–5. doi: 10.3201/eid1812.120929 (PMC3557876; doi:10.3201/eid1812.120929)
Supplement: Related texts

# Brief report: Enterovirus 71–associated Hand, Foot, and Mouth Disease, Southern Vietnam, 2011

**Authors:** Truong Huu Khanh, Saraswathy Sabanathan, Tran Tan Thanh, Le Phan Kim Thoa, Tang Chi Thuong, Vu thi Ty Hang, Jeremy Farrar, Tran Tinh Hien, Nguyen van Vinh Chau, H. Rogier van Doorn

**Affiliations:** Author affiliations: Children’s Hospital 1, Ho Chi Minh City, Vietnam (T.H. Khanh, L.P.K. Thoa, T.C. Thuong);; Oxford University Clinical Research Unit (S. Sabanathan, T.T. Thanh, V.t.T. Hang, J. Farrar, T.T. Hien, H.R. van Doorn);; Oxford University, Oxford, UK (S. Sabanathan, J. Farrar, T.T. Hien, H.R. van Doorn);; Hospital for Tropical Diseases, Ho Chi Minh City (N.v.V. Chau)

## Abstract

We prospectively studied 3,791 children hospitalized during 2011 during a large outbreak of enterovirus 71–associated hand, foot, and mouth disease in Vietnam. Formal assessment of public health interventions, use of intravenous immunoglobulin and other therapies, and factors predisposing for progression of disease is needed to improve clinical management.

In Southeast Asia, human enterovirus 71 (EV71) is a frequent cause of hand, foot, and mouth disease (HFMD) in Southeast Asia and resulting neurologic and cardiopulmonary complications. Children <5 years of age are at risk for symptomatic and severe disease, but the factors predisposing for severity are largely unknown.

In Vietnam, EV71 was first isolated in 2003. In 2005, an outbreak of HFMD was caused by an early peak of coxsackievirus A16 (CVA16), followed by a peak of EV71, associated with severe disease (*1*).

HFMD outbreaks occurring every 3 years have been reported from countries in the region to which it is endemic (*2*,*3*), but Vietnam had a high number of cases during February 2011–July 2012: a total of 174,677 cases (110,897 during 2011; 63,780 during the first 6 months of 2012) and 200 deaths were reported from Vietnam during this period. The outbreak peaked in week 38 (September 18–24, 2011, with ≈2,500 reported hospital admissions countrywide. Reported case-patients were mainly from southern Vietnam in 2011; in 2012, the outbreak spread to the northern provinces of Vietnam (*4*).

The Vietnamese Ministry of Health has implemented a clinical grading system to guide disease management. It is based on a grading system from Taiwan (*5*) and is a consensus of experienced physicians; it describes 4 grades of disease. Grade 1 is uncomplicated disease with fever and vesicles or papules on hands, feet, buttocks, and oral mucosa. In grade 2 disease, the central nervous system is involved, usually as myoclonus starting in the fingers. Grade 2 is further divided into grade 2a disease, when myoclonus is reported by the caregiver, and grade 2b disease, when myoclonus is observed by a physician. In grade 3 disease, autonomic dysfunction occurs with fever that is unresponsive to antipyretics and with hypertension and persistent tachycardia. Patients with grade 4 disease exhibit cardiopulmonary compromise with pulmonary edema or hemorrhage. Grades 2b, 3, and 4 describe severe disease and are indicators for hospital admission and treatment. Patients with grade 2a disease (reported myoclonus) also usually are admitted, and a small proportion of patients seeking care in the outpatient clinic with grade 1 disease are admitted for observation, on the basis of clinical judgment.

Myoclonus and more severe neurologic complications are treated with oral or intravenous phenobarbital. Heart rate, respiratory rate, blood pressure, and saturations are recorded to monitor progress of disease. When persistent tachycardia, fever unresponsive to antipyretics, irregular breathing, or persistent hypertension occur, intravenous immunoglobulin (IVIg) is administered. Children have arterial lines inserted for close observation of blood pressure. Hypertension is treated with milrinone, a phosphodiesterase inhibitor. When a child’s condition does not improve, hemofiltration is used in conjunction with full intensive care support as needed.

## The Study

Data were collected prospectively from 3,791 patients with HFMD in Children’s Hospital 1, Ho Chi Minh City, Vietnam, during September 18–November 30, 2011, of whom 2,364 (62%) were male. Patients’ median age was 20 months; 95% of children were 1–4 years of age (Figure 1). On admission, 647 (17%) patients were given the following grade 1; 2,750 (73%), grade 2a; 338 (9%), grade 2b; 42 (1%), grade 3; and 14 (0.4%), grade 4. Of all admissions, 159 (4%) had a maximum grade of 3 or 4 during their hospital course. Six 6 (0.2%) children died, of whom 2 had grade 4 disease, one had grade 3, two had grade 2b, and one had grade 2a. Median time to maximum grade was <24 hours; the median duration of hospitalization was 3 days (interquatrile range [IQR] 2–4) for all patients and 5 days (IQR 4–8) for patients who were admitted with grade 3 or 4 disease or whose illness progressed to that degree of severity.

**Figure 1:** Age distribution by 4-month interval of 3,667 children admitted to Children’s Hospital 1, Ho Chi Minh City, Vietnam, who had clinical diagnoses of hand, foot, and mouth disease, September 18–November 30, 2011. White bars indicate total number of cases; black bars indicate severe cases (grade 2b or worse). Severe cases are defined as grade 2b, 3, or 4 disease.

A total of 2,750 (73%) of the 3,791 patients were admitted with grade 2a HMFD; however, disease in only 121 (4%) progressed to grade 2b (94 children) or further (27 children), whereas disease in 75 (22%) of 338 children admitted with grade 2b progressed to grade 3 or 4. Disease in only 1 (0.2%) of 647 children admitted with grade 1 progressed to grade 3 or 4 (Table 1). A total of 443 (12%) children with severe cases of grade 2b or higher, were treated with IVIg, of which 22 (0.6%) were treated with hemofiltration.

**Table 1:** Hand, foot, and mouth disease grades at admission and grades to which disease progressed during hospitalization for 3,791 children, Children’s Hospital 1, Ho Chi Minh City, Vietnam, September 18–November 30, 2011

Grade at admission	Grade to which illness progressed, no. (%) cases					Total
	1	2a	2b	3	4	
1	640 (98.9)	0	6 (0.9)	1 (0.2)	0	647
2a		2,629 (95.6)	94 (3.4)	25 (0.9)	2 (0.1)	2,750
2b			263 (77.8)	71 (21.0)	4 (1.2)	338
3				38 (90.5)	4 (9.5)	42
4					14 (100.0)	14

Samples were analyzed with generic enterovirus reverse transcription PCR (RT-PCR) as described (*6*) and with EV71-specific real-time RT-PCR and CVA16-specific RT-PCR (Table 2) by using SuperScript One-Step RT-PCR reagents (Invitrogen, Carlsbad, CA, USA) under the following conditions: 50°C for 30 min, 95°C for 2 min, and 40 cycles of 95°C for 15 s, 55°C for 30 s, and 72°C for 20 s. Sequencing of viral protein (VP) 1 of EV71 was performed by using ABI Dye Terminator sequencing (Applied Biosystems, Foster City, CA, USA).

**Table 2:** Primers and probes used in study of EV71-associated hand, foot, and mouth disease, Children’s Hospital 1, Ho Chi Minh City, Vietnam, September 18–November 30, 2011*

Assay	Primer and probe	Sequence, 5′ → 3′	GenBank accession no.	PCR product size, bp
EV71 real-time RT-PCR	EV71-VP1-634F	GGAGAACACAARCARGAGAAAGA	AM490160.1	Real-time analysis
	EV71-VP1-743R	ACTAAAGGGTACTTGGACTTVGA		
	EV71-VP1-TaqMan	FAM-TGATGGGCACGTTCTCAGTGCG-BHQ1		
CVA16 RT-PCR	CoxA-VP1-526F	AACCCATCTGTGTTTGTGAAAA	JF317969.1	110
	CoxA-VP1-635R	CCGAAGGTGGGATAACCAT		
EV71 VP1 sequencing	EV71-VP1-3F	AGAYAGGGTGGCRGATGT	AM490160.1	701
	EV71-VP1-703R	CTGAGAACGTGCCCATCA		

*EV71, enterovirus 71; RT-PCR, reverse transcription PCR; VP, viral protein; FAM,  carboxyfluorescein; BHQ1, black hole quencher 1; CVA16, coxsackievirus A16; CoxA, coxsackievirus.

Virologic analysis was done on nose/throat or rectal swab specimens from 174 (33%) of 522 children with grade 2b disease or higher. A total of 132 (76%) of these were positive for by RT-PCR. CVA16 was not detected among these 174 children, and other enteroviruses were detected only sporadically.

In contrast to results from previous outbreaks in Vietnam, all of 11 sequences from this and other studies (*7*) showed that viruses belonged to subgenogroup C4, replacing C5 (Figure 2). Subgenogroup C4 has been found in the region since 1998 (*8*), and viruses from our study cluster with recent C4 viruses from the People’s Republic of China. Emergence of novel subgenotypes, often related to recombination events in noncoding regions (*9*), and switches of dominant subgenotypes are common and may be associated with large outbreaks. Most outbreaks reported in the Asia-Pacific region during the last decade were caused by previously undefined EV71 subgenogroups (*10*), but there is no evidence of differences in virulence. Data suggest cross-antigenicity among the different subgenotypes (*11*), i.e., EV71 constitutes 1 serotype.

**Figure 2:** Phylogenetic tree of enterovirus 71 viral protein 1 constructed by MEGA4 (www.megasoftware.net) with neighbor-joining method showing the relationship of 18 local sequences from 2010 and 2011 (triangles). Sequence names consist of the following information: the hospital at which the sample was obtained (HTD, Hospital for Tropical Diseases; CH1, Children’s Hospital 1; CH2, Children’s Hospital 2 (all from Ho Chi Minh City, Vietnam]); number in chronologic order/VNM for Vietnam/date (month-year). Reference genotypic sequences used in tree construction were obtained from GenBank. Detailed information about these sequences is available in reference (1).

## Conclusions

EV71 has emerged as a frequent cause of clinically severe HFMD and affects a large number of countries in the region. Although spreading locally, large epidemics with severe disease are confined to Southeast Asia. The potential for pandemic spread is unknown.

In Vietnam and surrounding countries, EV71 has become endemic, and seroprevalence studies show a high force of infection with a seroconversion rate of up to 14% during the second year of life in southern Vietnam (*12*). The case-fatality rate in this and other outbreaks is generally low (<0.5%) (*13*), but the large number of cases and relative absence of prognostic factors for progression to more severe disease considerably affect the health care system, requiring monitoring and observation of large numbers of patients.

This study included all patients hospitalized during September 18–November 30, 2011, in the largest children’s referral hospital in southern Vietnam. The study’s limitations are as follows: because observations are only from hospitalized patients in 1 hospital, the study did not include all cases of severe disease in southern Vietnam or any outpatients. In addition, virologic testing was available only for patients with severe disease and only for one third of those.

Until a vaccine becomes available, control of EV71 is limited to promotion of public health interventions, such as hand washing, exclusion of ill children from school settings, and improved clinical management of EV71-associated HFMD. During the EV71 outbreak in southern Vietnam during 2011–2012, most children (90% in this study) with HFMD were hospitalized with mild disease (grade 2a or below), and more severe disease (grade 2b or higher) developed in only a small fraction (4% in this study) of these patients. To improve clinical management and reduce the strain on the health care system, formal assessment of public health interventions and use of IVIg and other therapeutic options and of factors predisposing the patient for progression of disease is needed to improve clinical management and reduce the strain on the health care system.

## References

[1] Tu PV, Thao NT, Perera D, Huu TK, Tien NT, Thuong TC, Epidemiologic and virologic investigation of hand, foot, and mouth disease, southern Vietnam, 2005. Emerg Infect Dis. 2007;13:1733–41. 10.3201/eid1311.07063218217559PMC3375788

[2] Mizuta K, Abiko C, Murata T, Matsuzaki Y, Itagaki T, Sanjoh K, Frequent importation of enterovirus 71 from surrounding countries into the local community of Yamagata, Japan, between 1998 and 2003. J Clin Microbiol. 2005;43:6171–5. 10.1128/JCM.43.12.6171-6175.200516333123PMC1317214

[3] Ooi MH, Wong SC, Podin Y, Akin W, del Sel S, Mohan A, Human enterovirus 71 disease in Sarawak, Malaysia: a prospective clinical, virological, and molecular epidemiological study. Clin Infect Dis. 2007;44:646–56. 10.1086/51107317278054

[4] World Health Organization. Hand, foot and mouth disease situation update, 9 August 2012. [cited 2012 Aug 10]. http://www.wpro.who.int/emerging_diseases/HFMD/en/index.html

[5] Huang CC, Liu CC, Chang YC, Chen CY, Wang ST, Yeh TF. Neurologic complications in children with enterovirus 71 infection. N Engl J Med. 1999;341:936–42. 10.1056/NEJM19990923341130210498488

[6] Beld M, Minnaar R, Weel J, Sol C, Damen M, van der Avoort H, Highly sensitive assay for detection of enterovirus in clinical specimens by reverse transcription–PCR with an armored RNA internal control. J Clin Microbiol. 2004;42:3059–64. 10.1128/JCM.42.7.3059-3064.200415243060PMC446274

[7] Thao NT. Hand, foot and mouth disease—Viet Nam (03): Ho Chi Minh City. ProMed. 2011 Jun 6 [cited 2012 Aug 1]. http://www.promedmail.org, archive no. 20110607.1739.

[8] Lin KH, Hwang KP, Ke GM, Wang CF, Ke LY, Hsu YT, Evolution of EV71 genogroup in Taiwan from 1998 to 2005: an emerging of subgenogroup C4 of EV71. J Med Virol. 2006;78:254–62. 10.1002/jmv.2053416372302

[9] McWilliam Leitch EC, Cabrerizo M, Cardosa J, Harvala H, Ivanova OE, Koike S, The association of recombination events in the founding and emergence of subgenogroup evolutionary lineages of human enterovirus 71. J Virol. 2012;86:2676–85. 10.1128/JVI.06065-1122205739PMC3302253

[10] Tee KK, Lam TT, Chan YF, Bible JM, Kamarulzaman A, Tong CY, Evolutionary genetics of human enterovirus 71: origin, population dynamics, natural selection, and seasonal periodicity of the VP1 gene. J Virol. 2010;84:3339–50. 10.1128/JVI.01019-0920089660PMC2838098

[11] Mizuta K, Aoki Y, Suto A, Ootani K, Katsushima N, Itagaki T, Cross-antigenicity among EV71 strains from different genogroups isolated in Yamagata, Japan, between 1990 and 2007. Vaccine. 2009;27:3153–8. 10.1016/j.vaccine.2009.03.06019446185

[12] Tran CB, Nguyen HT, Phan HT, Tran NV, Wills B, Farrar J, The seroprevalence and seroincidence of enterovirus71 infection in infants and children in Ho Chi Minh City, Viet Nam. PLoS ONE. 2011;6:e21116. 10.1371/journal.pone.002111621765891PMC3134465

[13] Lu CY, Lee CY, Kao CL, Shao WY, Lee PI, Twu SJ, Incidence and case-fatality rates resulting from the 1998 enterovirus 71 outbreak in Taiwan. J Med Virol. 2002;67:217–23. 10.1002/jmv.221011992582

# Brief report: Enterovirus 71–associated Hand, Foot, and Mouth Disease, Southern Vietnam, 2011

**Authors:** Truong Huu Khanh, Saraswathy Sabanathan, Tran Tan Thanh, Le Phan Kim Thoa, Tang Chi Thuong, Vu thi Ty Hang, Jeremy Farrar, Tran Tinh Hien, Nguyen van Vinh Chau, H. Rogier van Doorn

**Affiliations:** Author affiliations: Children’s Hospital 1, Ho Chi Minh City, Vietnam (T.H. Khanh, L.P.K. Thoa, T.C. Thuong);; Oxford University Clinical Research Unit (S. Sabanathan, T.T. Thanh, V.t.T. Hang, J. Farrar, T.T. Hien, H.R. van Doorn);; Oxford University, Oxford, UK (S. Sabanathan, J. Farrar, T.T. Hien, H.R. van Doorn);; Hospital for Tropical Diseases, Ho Chi Minh City (N.v.V. Chau)

## Medscape CME Quiz

## Earning CME Credit

To obtain credit, you should first read the journal article. After reading the article, you should be able to answer the following, related, multiple-choice questions. To complete the questions (with a minimum 70% passing score) and earn continuing medical education (CME) credit, please go to www.medscape.org/journal/eid. Credit cannot be obtained for tests completed on paper, although you may use the worksheet below to keep a record of your answers. You must be a registered user on Medscape.org. If you are not registered on Medscape.org, please click on the New Users: Free Registration link on the left hand side of the website to register. Only one answer is correct for each question. Once you successfully answer all post-test questions you will be able to view and/or print your certificate. For questions regarding the content of this activity, contact the accredited provider, CME@medscape.net. For technical assistance, contact CME@webmd.net. American Medical Association’s Physician’s Recognition Award (AMA PRA) credits are accepted in the US as evidence of participation in CME activities. For further information on this award, please refer to http://www.ama-assn.org/ama/pub/category/2922.html. The AMA has determined that physicians not licensed in the US who participate in this CME activity are eligible for *AMA PRA Category 1 Credits*™. Through agreements that the AMA has made with agencies in some countries, AMA PRA credit may be acceptable as evidence of participation in CME activities. If you are not licensed in the US, please complete the questions online, print the certificate and present it to your national medical association for review.

## Article Title:  Enterovirus 71–associated Hand, Foot, and Mouth Disease, Southern Vietnam, 2011

## CME Questions

1. You are seeing a 3-year-old girl with a two-day history of fever. She developed oral ulcers this morning, and you suspect that she has hand, foot, and mouth disease (HFMD). In the current study, what virus type was associated with the majority of cases of HFMD requiring hospitalization? 

A. Enterovirus 71

B. Coxsackievirus A16

C. Echovirus 13

D. Poliovirus 2

2. The patient is treated with supportive care but returns to your clinic the next day when her mother notes myoclonus in her fingers. While the patient still has fever and a rash, there are no other symptoms or physical signs. According to the clinical grading system used in the current study, what grade of HFMD does this patient now have?

A. Grade 1

B. Grade 2

C. Grade 3

D. Grade 4

3. What should you consider regarding the clinical presentation of cases of HFMD in the current study?

A. Most children were over 5 years old 

B. The mortality rate exceeded 10%

C. The average time for progression to most severe illness was 5 days

D. Children with caregiver-reported myoclonus had a low rate of clinical progression 

4. The patient goes on to develop multiple complications of HFMD. What treatments were used for the associated complications in the current study?

A. Myoclonus — diazepam 

B. Seizures — carbamazepine

C. Fever unresponsive to antipyretics — IVIg

D. Hypertension — beta blockers

## Activity Evaluation

**Table:** 

**1. The activity supported the learning objectives.**				
Strongly Disagree				Strongly Agree
1	2	3	4	5
**2. The material was organized clearly for learning to occur.**				
Strongly Disagree				Strongly Agree
1	2	3	4	5
**3. The content learned from this activity will impact my practice.**				
Strongly Disagree				Strongly Agree
1	2	3	4	5
**4. The activity was presented objectively and free of commercial bias.**				
Strongly Disagree				Strongly Agree
1	2	3	4	5
